# Bis(pyridine-3-carboxylic acid-κ*N*)silver(I) perchlorate

**DOI:** 10.1107/S1600536808044206

**Published:** 2009-01-08

**Authors:** Xiao-Yan Nie, Qian-Zhu Li

**Affiliations:** a Department of Chemistry, Bijie University, Bijie 551700, People’s Republic of China

## Abstract

In the title compound, [Ag(C_6_H_5_NO_2_)_2_]ClO_4_, the Ag^I^ atom shows an almost linear coordination geometry, defined by two N atoms from two pyridine-3-carboxylic acid ligands. The complex cations are linked by hydrogen bonds between the carboxyl groups into a chain. The chains are further connected through C—H⋯O hydrogen bonds and a weak Ag⋯O inter­action [2.757 (8) Å] into a layer. Another Ag⋯O inter­action [2.899 (2) Å] and a C—H⋯O hydrogen bond connect the layers into a three-dimensional network.

## Related literature

For general background on coordination polymers and open-framework materials, see: James (2003 ▶); Serre *et al.* (2004 ▶); Yaghi *et al.* (1998 ▶, 2003 ▶). For related structures, see: Evans & Lin (2001 ▶); Luo *et al.* (2004 ▶).
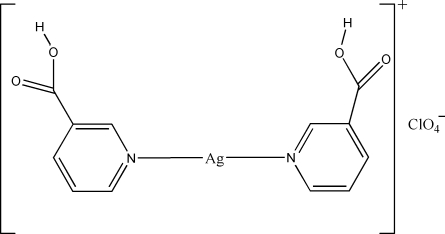


## Experimental

### 

#### Crystal data

[Ag(C_6_H_5_NO_2_)_2_]ClO_4_
*M* *_r_* = 453.54
Monoclinic, 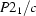

*a* = 8.0139 (4) Å
*b* = 26.3288 (15) Å
*c* = 7.6891 (4) Å
β = 110.728 (1)°
*V* = 1517.36 (14) Å^3^
*Z* = 4
Mo *K*α radiation
μ = 1.55 mm^−1^
*T* = 273 (2) K
0.29 × 0.25 × 0.21 mm

#### Data collection

Bruker APEXII CCD diffractometer
Absorption correction: multi-scan (*SADABS*; Sheldrick, 1996 ▶) *T*_min_ = 0.649, *T*_max_ = 0.731
7763 measured reflections
2729 independent reflections
2150 reflections with *I* > 2σ(*I*)
*R*_int_ = 0.029

#### Refinement

*R*[*F* ^2^ > 2σ(*F* ^2^)] = 0.043
*wR*(*F* ^2^) = 0.123
*S* = 0.87
2729 reflections
219 parameters
H-atom parameters constrained
Δρ_max_ = 1.26 e Å^−3^
Δρ_min_ = −0.57 e Å^−3^

### 

Data collection: *APEX2* (Bruker, 2007 ▶); cell refinement: *SAINT* (Bruker, 2007 ▶); data reduction: *SAINT*; program(s) used to solve structure: *SHELXS97* (Sheldrick, 2008 ▶); program(s) used to refine structure: *SHELXL97* (Sheldrick, 2008 ▶); molecular graphics: *SHELXTL* (Sheldrick, 2008 ▶); software used to prepare material for publication: *SHELXTL*.

## Supplementary Material

Crystal structure: contains datablocks I, global. DOI: 10.1107/S1600536808044206/hy2176sup1.cif

Structure factors: contains datablocks I. DOI: 10.1107/S1600536808044206/hy2176Isup2.hkl

Additional supplementary materials:  crystallographic information; 3D view; checkCIF report

## Figures and Tables

**Table:** 

Ag1—N1	2.178 (4)
Ag1—N2	2.185 (4)

**Table:** 

N1—Ag1—N2	165.65 (15)

**Table 2:** Hydrogen-bond geometry (Å, °)

*D*—H⋯*A*	*D*—H	H⋯*A*	*D*⋯*A*	*D*—H⋯*A*
C1—H1⋯O5^i^	0.93	2.51	3.244 (8)	136
C4—H4⋯O7^ii^	0.93	2.52	3.266 (8)	139
C6—H6⋯O7	0.93	2.52	3.248 (8)	136
C7—H7⋯O8^i^	0.93	2.49	3.287 (9)	144
C12—H12⋯O7	0.93	2.38	3.228 (9)	152
O2—H2⋯O4^iii^	0.82	1.84	2.649 (5)	169
O3—H3⋯O1^iv^	0.82	1.87	2.689 (5)	175

Symmetry codes: (i) 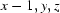; (ii) 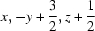; (iii) 
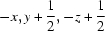
; (iv) 
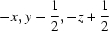
.

**Fig. 1.**
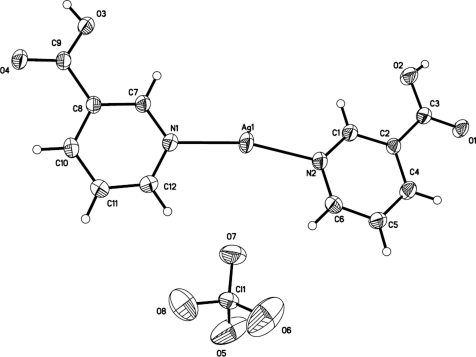
The structure of the title compound. Displacement ellipsoids are drawn at the 30% probability level.

**Fig. 2.**
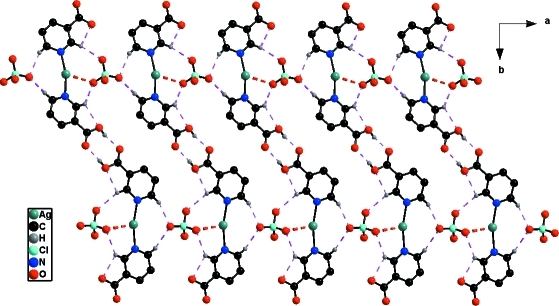
The layer in the title compound. Hydrogen bonds and weak Ag···O interactions are shown as dashed lines.

## supplementary crystallographic information

### Comment

The use of multifunctional organic linker molecules in the preparation of coordination polymers and open-framework materials has led to the development of a rich field of chemistry owing to the potential applications of these materials in catalysis, separation, gas storage and molecular recognition (James, 2003; Serre *et al.*, 2004; Yaghi *et al.*, 1998, 2003). In our investigations we used nicotinic acid ligands for the preparation of new coordination polymers, because it can act as a multidentate ligand with versatile binding and coordination modes (Evans & Lin, 2001; Luo *et al.*, 2004). In this paper, we report the crystal structure of the title compound, a new Ag^I^ complex obtained by the reaction of nicotinic acid, AgNO_3_ and perchloric acid in water.

As shown in Fig. 1, the title compound consists of a [Ag(C_6_H_5_NO_2_)_2_]^+^ cation and a perchlorate anion. The Ag^I^ atom exhibits a linear coordination geometry, defined by two N atoms from two pyridine-3-carboxylic acid ligands (Table 1). The complex cations are linked by hydrogen bonds between the carboxyl groups into a chain (Table 2). The chains are further connected by C—H···O hydrogen bonds involving C1, C6, C7 and C12 atoms and the perchlorate anions, and by a weak Ag1···O5(x-1, y, z) interaction [2.757 (8) Å] into a layer (Fig. 2). Another Ag1···O1(x, 3/2-y, z-1/2) interaction [2.899 (2) Å] and a C4—H4···O7(x, 3/2-y, 1/2+z) hydrogen bond connect the layers into a three-dimensional network.

### Experimental

A mixture of AgNO_3_ (0.169 g, 1 mmol), perchloric acid (0.12 ml), nicotinic acid (0.123 g, 1 mmol) and H_2_O (10 ml) was placed in a 23 ml Teflon-lined reactor, which was heated to 433 K for 3 d and then cooled to room temperature at a rate of 10 K h^-1^. The crystals obtained were washed with water and dried in air.

### Refinement

H atoms on C atoms were positioned geometrically and refined as riding atoms, with C—H = 0.93 Å and *U*_iso_(H) = 1.2*U*_eq_(C). H atoms on O atoms were located in difference Fourier maps and were fixed with O—H = 0.82 Å and *U*_iso_(H) = 1.5*U*_eq_(O). The highest residual electron density was found 1.31 Å from atom O7 and the deepest hole 0.46 Å from atom O7.

### Crystal data

**Table:**

[Ag(C_6_H_5_NO_2_)_2_]ClO_4_	*F*(000) = 896
*M**_r_* = 453.54	*D*_x_ = 1.985 Mg m^−^^3^
Monoclinic, *P*2_1_/*c*	Mo *K*α radiation, λ = 0.71073 Å
Hall symbol: -P 2ybc	Cell parameters from 5837 reflections
*a* = 8.0139 (4) Å	θ = 2.8–27.9°
*b* = 26.3288 (15) Å	µ = 1.55 mm^−^^1^
*c* = 7.6891 (4) Å	*T* = 273 K
β = 110.728 (1)°	Block, colourless
*V* = 1517.36 (14) Å^3^	0.29 × 0.25 × 0.21 mm
*Z* = 4	

### Data collection

**Table:**

Bruker APEXII CCD diffractometer	2729 independent reflections
Radiation source: fine-focus sealed tube	2150 reflections with *I* > 2σ(*I*)
graphite	*R*_int_ = 0.029
φ and ω scans	θ_max_ = 25.2°, θ_min_ = 1.6°
Absorption correction: multi-scan (*SADABS*; Sheldrick, 1996)	*h* = −8→9
*T*_min_ = 0.649, *T*_max_ = 0.731	*k* = −29→31
7763 measured reflections	*l* = −9→8

### Refinement

**Table:**

Refinement on *F*^2^	Primary atom site location: structure-invariant direct methods
Least-squares matrix: full	Secondary atom site location: difference Fourier map
*R*[*F*^2^ > 2σ(*F*^2^)] = 0.043	Hydrogen site location: inferred from neighbouring sites
*wR*(*F*^2^) = 0.123	H-atom parameters constrained
*S* = 0.87	*w* = 1/[σ^2^(*F*_o_^2^) + (0.0736*P*)^2^ + 4.5241*P*] where *P* = (*F*_o_^2^ + 2*F*_c_^2^)/3
2729 reflections	(Δ/σ)_max_ = 0.001
219 parameters	Δρ_max_ = 1.26 e Å^−^^3^
0 restraints	Δρ_min_ = −0.57 e Å^−^^3^

### Fractional atomic coordinates and isotropic or equivalent isotropic displacement parameters (Å^2^)

**Table:**

	*x*	*y*	*z*	*U*_iso_*/*U*_eq_	
Ag1	0.40344 (6)	0.612387 (13)	0.34075 (6)	0.05534 (19)	
C1	0.3293 (6)	0.72596 (17)	0.4083 (7)	0.0438 (11)	
H1	0.2115	0.7150	0.3691	0.053*	
Cl1	0.97387 (18)	0.61558 (5)	0.3633 (2)	0.0544 (3)	
N1	0.4208 (5)	0.53278 (14)	0.2719 (6)	0.0457 (9)	
O1	0.2535 (4)	0.86018 (12)	0.4508 (5)	0.0488 (8)	
C2	0.3650 (6)	0.77700 (16)	0.4460 (6)	0.0367 (10)	
N2	0.4580 (5)	0.69166 (14)	0.4261 (6)	0.0437 (9)	
O2	0.0616 (5)	0.79535 (13)	0.3776 (6)	0.0596 (10)	
H2	−0.0120	0.8184	0.3499	0.089*	
C3	0.2204 (6)	0.81428 (17)	0.4237 (7)	0.0425 (11)	
O3	0.0023 (4)	0.43163 (13)	0.1274 (6)	0.0541 (9)	
H3	−0.0738	0.4093	0.0982	0.081*	
C4	0.5421 (6)	0.79317 (18)	0.5073 (7)	0.0466 (11)	
H4	0.5706	0.8271	0.5356	0.056*	
O4	0.1889 (5)	0.36546 (12)	0.1767 (5)	0.0519 (9)	
C5	0.6742 (6)	0.75790 (19)	0.5251 (7)	0.0488 (12)	
H5	0.7932	0.7678	0.5642	0.059*	
O5	1.0454 (10)	0.6352 (3)	0.2338 (8)	0.147 (3)	
C6	0.6276 (6)	0.70794 (18)	0.4842 (7)	0.0455 (11)	
H6	0.7176	0.6843	0.4974	0.055*	
O6	1.0605 (13)	0.6412 (4)	0.5267 (9)	0.174 (4)	
C7	0.2868 (6)	0.49924 (17)	0.2338 (7)	0.0409 (10)	
H7	0.1745	0.5107	0.2256	0.049*	
O7	0.7884 (7)	0.6209 (2)	0.2885 (11)	0.117 (2)	
C8	0.3093 (6)	0.44812 (16)	0.2063 (6)	0.0372 (10)	
O8	1.0104 (10)	0.5643 (3)	0.3779 (16)	0.187 (4)	
C9	0.1598 (6)	0.41126 (17)	0.1687 (7)	0.0409 (10)	
C10	0.4758 (7)	0.43088 (19)	0.2185 (8)	0.0500 (12)	
H10	0.4948	0.3966	0.2025	0.060*	
C11	0.6130 (7)	0.4653 (2)	0.2546 (8)	0.0567 (14)	
H11	0.7258	0.4547	0.2613	0.068*	
C12	0.5813 (7)	0.5153 (2)	0.2807 (8)	0.0534 (13)	
H12	0.6751	0.5383	0.3056	0.064*	

### Atomic displacement parameters (Å^2^)

**Table:**

	*U*^11^	*U*^22^	*U*^33^	*U*^12^	*U*^13^	*U*^23^
Ag1	0.0591 (3)	0.0284 (2)	0.0765 (3)	0.00062 (16)	0.0214 (2)	−0.00512 (17)
C1	0.039 (2)	0.035 (2)	0.055 (3)	−0.0009 (19)	0.014 (2)	−0.005 (2)
Cl1	0.0416 (7)	0.0587 (8)	0.0659 (8)	0.0030 (6)	0.0228 (6)	0.0001 (6)
N1	0.044 (2)	0.033 (2)	0.060 (3)	−0.0025 (17)	0.0185 (19)	−0.0041 (18)
O1	0.0469 (19)	0.0275 (17)	0.067 (2)	−0.0023 (14)	0.0137 (17)	0.0003 (15)
C2	0.041 (2)	0.030 (2)	0.040 (2)	−0.0015 (18)	0.0161 (19)	−0.0009 (18)
N2	0.044 (2)	0.032 (2)	0.054 (2)	0.0018 (17)	0.0163 (18)	−0.0036 (17)
O2	0.043 (2)	0.0321 (18)	0.100 (3)	0.0010 (15)	0.020 (2)	−0.0021 (18)
C3	0.044 (3)	0.034 (2)	0.050 (3)	−0.003 (2)	0.017 (2)	−0.002 (2)
O3	0.0402 (19)	0.0365 (18)	0.084 (3)	−0.0042 (15)	0.0195 (18)	−0.0055 (18)
C4	0.047 (3)	0.035 (2)	0.054 (3)	−0.005 (2)	0.014 (2)	0.000 (2)
O4	0.049 (2)	0.0302 (18)	0.074 (2)	0.0013 (15)	0.0181 (17)	0.0021 (16)
C5	0.038 (3)	0.042 (3)	0.065 (3)	−0.002 (2)	0.016 (2)	−0.002 (2)
O5	0.133 (5)	0.226 (9)	0.086 (4)	−0.095 (6)	0.043 (4)	−0.031 (5)
C6	0.042 (3)	0.043 (3)	0.051 (3)	0.006 (2)	0.016 (2)	0.000 (2)
O6	0.219 (9)	0.219 (9)	0.081 (4)	−0.109 (8)	0.050 (5)	−0.050 (5)
C7	0.036 (2)	0.033 (2)	0.051 (3)	0.0015 (18)	0.012 (2)	−0.0008 (19)
O7	0.061 (3)	0.086 (4)	0.210 (7)	0.007 (3)	0.055 (4)	0.005 (4)
C8	0.038 (2)	0.032 (2)	0.041 (2)	0.0006 (18)	0.0116 (19)	−0.0027 (18)
O8	0.118 (6)	0.104 (5)	0.323 (12)	0.054 (4)	0.057 (7)	−0.004 (7)
C9	0.042 (3)	0.031 (2)	0.047 (3)	0.0009 (19)	0.013 (2)	−0.0031 (19)
C10	0.052 (3)	0.034 (3)	0.064 (3)	0.004 (2)	0.021 (2)	−0.008 (2)
C11	0.044 (3)	0.051 (3)	0.079 (4)	0.001 (2)	0.027 (3)	−0.010 (3)
C12	0.043 (3)	0.045 (3)	0.075 (4)	−0.005 (2)	0.024 (3)	−0.004 (3)

### Geometric parameters (Å, °)

**Table:**

Ag1—N1	2.178 (4)	O2—H2	0.8200
Ag1—N2	2.185 (4)	O3—C9	1.303 (6)
Ag1—O1^i^	2.899 (2)	O3—H3	0.8200
Ag1—O5^ii^	2.757 (8)	C4—C5	1.378 (7)
C1—N2	1.341 (6)	C4—H4	0.9300
C1—C2	1.383 (6)	O4—C9	1.226 (6)
C1—H1	0.9300	C5—C6	1.373 (7)
Cl1—O8	1.378 (7)	C5—H5	0.9300
Cl1—O6	1.378 (6)	C6—H6	0.9300
Cl1—O7	1.398 (6)	C7—C8	1.384 (6)
Cl1—O5	1.411 (6)	C7—H7	0.9300
N1—C7	1.341 (6)	C8—C10	1.381 (7)
N1—C12	1.345 (6)	C8—C9	1.489 (6)
O1—C3	1.239 (5)	C10—C11	1.375 (7)
C2—C4	1.394 (6)	C10—H10	0.9300
C2—C3	1.482 (6)	C11—C12	1.370 (7)
N2—C6	1.342 (6)	C11—H11	0.9300
O2—C3	1.294 (6)	C12—H12	0.9300
			
N1—Ag1—N2	165.65 (15)	C2—C4—H4	120.6
N2—C1—C2	122.6 (4)	C6—C5—C4	119.1 (5)
N2—C1—H1	118.7	C6—C5—H5	120.5
C2—C1—H1	118.7	C4—C5—H5	120.5
O8—Cl1—O6	112.4 (6)	N2—C6—C5	122.9 (4)
O8—Cl1—O7	107.2 (4)	N2—C6—H6	118.5
O6—Cl1—O7	116.4 (6)	C5—C6—H6	118.5
O8—Cl1—O5	106.8 (6)	N1—C7—C8	122.4 (4)
O6—Cl1—O5	105.3 (4)	N1—C7—H7	118.8
O7—Cl1—O5	108.1 (5)	C8—C7—H7	118.8
C7—N1—C12	117.6 (4)	C10—C8—C7	119.0 (4)
C7—N1—Ag1	124.9 (3)	C10—C8—C9	119.4 (4)
C12—N1—Ag1	117.3 (3)	C7—C8—C9	121.6 (4)
C1—C2—C4	118.5 (4)	O4—C9—O3	124.6 (4)
C1—C2—C3	121.6 (4)	O4—C9—C8	120.4 (4)
C4—C2—C3	119.9 (4)	O3—C9—C8	115.0 (4)
C1—N2—C6	118.1 (4)	C11—C10—C8	118.8 (5)
C1—N2—Ag1	123.2 (3)	C11—C10—H10	120.6
C6—N2—Ag1	118.5 (3)	C8—C10—H10	120.6
C3—O2—H2	109.5	C12—C11—C10	119.1 (5)
O1—C3—O2	123.6 (4)	C12—C11—H11	120.4
O1—C3—C2	120.9 (4)	C10—C11—H11	120.4
O2—C3—C2	115.4 (4)	N1—C12—C11	123.0 (5)
C9—O3—H3	109.5	N1—C12—H12	118.5
C5—C4—C2	118.8 (4)	C11—C12—H12	118.5
C5—C4—H4	120.6		

Symmetry codes: (i) *x*, −*y*+3/2, *z*−1/2; (ii) *x*−1, *y*, *z*.

### Hydrogen-bond geometry (Å, °)

**Table:**

*D*—H···*A*	*D*—H	H···*A*	*D*···*A*	*D*—H···*A*
C1—H1···O5^ii^	0.93	2.51	3.244 (8)	136
C4—H4···O7^iii^	0.93	2.52	3.266 (8)	139
C6—H6···O7	0.93	2.52	3.248 (8)	136
C7—H7···O8^ii^	0.93	2.49	3.287 (9)	144
C12—H12···O7	0.93	2.38	3.228 (9)	152
O2—H2···O4^iv^	0.82	1.84	2.649 (5)	169
O3—H3···O1^v^	0.82	1.87	2.689 (5)	175

Symmetry codes: (ii) *x*−1, *y*, *z*; (iii) *x*, −*y*+3/2, *z*+1/2; (iv) −*x*, *y*+1/2, −*z*+1/2; (v) −*x*, *y*−1/2, −*z*+1/2.
